# Supplementary material for: An instant messaging mobile phone application for promoting HIV pre-exposure prophylaxis uptake among Chinese gay, bisexual and other men who have sex with men: A mixed methods feasibility and piloting randomized controlled trial study
Source: PLoS One. 2023 Nov 13;18(11):e0285036. doi: 10.1371/journal.pone.0285036 (PMC10642832; doi:10.1371/journal.pone.0285036)
Supplement: S1 Table — (DOCX) [file pone.0285036.s002.docx]

**S2 Table. Summary of the PrEP mini-app key functions**

| Key functions | Intervention  objectives | Intervention strategies | | | |
| --- | --- | --- | --- | --- | --- |
|  |  | Information | Motivation | Behavioral Skills | Mental Health |
| Mini-classroom | Build knowledge and skills around local HIV care system, enhance interest and motivation to use PrEP, and increase self-efficacy in HIV/STI prevention strategies; Improve participants’ mental health management skills. | Educational materials in multimedia forms, including text, videos, and graphics. | Real stories of PrEP users;  Positive meanings of using PrEP and HIV/STI testing. | List local PrEP and other HIV/STI care providers and contact information; Tips of how to have safe sex and condom use; Tips of PrEP initiation, adherence, and management. | Links to local support groups and mental health resources; Self-management for mental health; How to deal with stigma and discrimination against LGBTQ community. |
| Online chat | Enable GBMSM to describe their feelings or concerns related to HIV, sexual health, or this intervention study, and help them make healthy decisions. | Answer HIV/sexual health-related questions and provide additional information if needed. | Tailored health advice regarding PrEP use. | Referral to the study’s HIV/PrEP clinic, or other healthcare providers based on individual needs | Listen to participants’ needs, and refer to local support groups or mental health care resources, as needed. |
| Online HIV/syphilis self-test kit ordering | Establish an individual habit of routine testing for HIV and syphilis.  Reduce structural and psychosocial barriers to HIV/syphilis testing. | Information about how to complete the home-based test kit. | Cue to action and removes barriers of in-person testing. | An HIV/syphilis home-based test kit ordering system. | N/A |
| User profile center | Allow participants to keep track of their HIV/syphilis testing behaviors. | N/A | N/A | A user profile page where users can manage orders of HIV/syphilis test kits and keep a record of test results. | N/A |
