# Supplementary material for: An instant messaging mobile phone application for promoting HIV pre-exposure prophylaxis uptake among Chinese gay, bisexual and other men who have sex with men: A mixed methods feasibility and piloting randomized controlled trial study
Source: PLoS One. 2023 Nov 13;18(11):e0285036. doi: 10.1371/journal.pone.0285036 (PMC10642832; doi:10.1371/journal.pone.0285036)
Supplement: S2 Table — (DOCX) [file pone.0285036.s003.docx]

**S3 Table. Characteristics of Interviewed Participants**

| ID | Study Arm | PrEP Cascade at the end of intervention | App use  (Intervention only) | Age | Number of interviews |
| --- | --- | --- | --- | --- | --- |
| 2 | Mini-app | None | High | 24 | 1 |
| 5 | Mini-app | None | Low | 26 | 2 |
| 12 | Mini-app | Filled PrEP once & completed 2-month clinical follow-up visit | Low | 26 | 2 |
| 13 | Mini-app | Filled PrEP once | High | 30 | 2 |
| 22 | Mini-app | None | High | 20 | 2 |
| 23 | Mini-app | Filled PrEP once | Low | 31 | 2 |
| 26 | Mini-app | Received initial lab tests only | Low | 28 | 2 |
| 29 | Mini-app | Received initial prescription only | High | 25 | 2 |
| 46 | Mini-app | Filled PrEP twice & completed 2-month clinical follow-up visit | High | 28 | 2 |
| 48 | Mini-app | None | Low | 30 | 2 |
| 55 | Mini-app | None | Low | 24 | 2 |
| 68 | Mini-app | Filled PrEP once | Low | 28 | 2 |
| 4 | Control | Received initial lab tests only | N/A | 29 | 2 |
| 16 | Control | Filled PrEP once & completed 2-month clinical follow-up visit | N/A | 36 | 2 |
| 27 | Control | Filled PrEP twice & completed 2-month clinical follow-up visit | N/A | 30 | 1 |
| 38 | Control | Filled PrEP once | N/A | 26 | 1 |
| 67 | Control | Filled PrEP twice & completed 2-month clinical follow-up visit | N/A | 32 | 1 |
| 70 | Control | None | N/A | 31 | 2 |
