# Supplementary material for: An instant messaging mobile phone application for promoting HIV pre-exposure prophylaxis uptake among Chinese gay, bisexual and other men who have sex with men: A mixed methods feasibility and piloting randomized controlled trial study
Source: PLoS One. 2023 Nov 13;18(11):e0285036. doi: 10.1371/journal.pone.0285036 (PMC10642832; doi:10.1371/journal.pone.0285036)
Supplement: S3 Table — (DOCX) [file pone.0285036.s004.docx]

**S4 Table. System Usability Scale Scores**

| Item | Median | Mean | SD |
| --- | --- | --- | --- |
| 1. I think that I would like to use this mini-app frequently. | 2 | 2.11 | 0.88 |
| 2. I thought the mini-app was easy to use. | 3 | 3.2 | 0.56 |
| 3. I found the various functions in this mini-app were well integrated. | 3 | 2.86 | 0.64 |
| 4. I would imagine that most people would learn to use this mini-app very quickly. | 3 | 2.98 | 0.67 |
| 5. I felt very confident using the mini-app. | 3 | 2.88 | 0.73 |
| 6. I found the mini-app unnecessarily complex. | 2 | 1.91 | 0.72 |
| 7. I think that I would need the support of a technical person to be able to use this mini-app. | 1 | 1.16 | 0.92 |
| 8. I thought there was too much inconsistency in this mini-app. | 1 | 1.35 | 0.92 |
| 9. I found the mini-app very cumbersome to use. | 1 | 1.30 | 0.89 |
| 10. I needed to learn a lot of things before I could get going with this mini-app. | 1 | 1.05 | 0.79 |

NOTE: Measured at Week 8 (n=43, 0=strongly disagree, 1=disagree, 2=neutral, 3=agree, 4=strongly agree)
