# Supplementary material for: An instant messaging mobile phone application for promoting HIV pre-exposure prophylaxis uptake among Chinese gay, bisexual and other men who have sex with men: A mixed methods feasibility and piloting randomized controlled trial study
Source: PLoS One. 2023 Nov 13;18(11):e0285036. doi: 10.1371/journal.pone.0285036 (PMC10642832; doi:10.1371/journal.pone.0285036)
Supplement: S1 File — (DOCX) [file pone.0285036.s005.docx]

**The Development of a Mobile-based App to Increase Uptake of Pre-Exposure Prophylaxis (PrEP) by Men Who Have Sex with Men in China**

Funding Sponsor: NIH National Institute of Allergy and Infectious Diseases (NIAID)

Funding Mechanism: R01- AI114310-S1

| **Protocol Date: Sept 2, 2020** |  |
| --- | --- |
| **Protocol Version: V3** |  |
| **Study Sponsor and Principal Investigator:** | Chunyan Li, MSc  Graduate Research Assistant  Address: 135 Dauer Dr, 302 Rosenau Hall, Chapel Hill, NC 27599  919-966-6695 (PHONE)  E-mail: [chunyli@live.unc.edu](mailto:chunyli@live.unc.edu) |
| Co-Investigator(s): | Kathryn Muessig, PhD  Associate Professor  Address: 306 Rosenau Hall  CB# 7440  Chapel Hill, NC 27599  Email: [kmuessig@med.unc.edu](mailto:kmuessig@med.unc.edu)  Joseph D. Tucker, MD, PhD  Associate Professor  Address: 130 Mason Farm Road, CB#7030, Chapel Hill, NC 27514  E-mail: [jdtuker@med.unc.edu](mailto:jdtuker@med.unc.edu) |
|  | Linghua Li, MD  Professor  Address: 627 Dongfeng Dong Road, Yuexiu District, Guangzhou, Guangdong, 510050, China,  +96-13725297174(PHONE)  [llheliza@126.com](mailto:llheliza@126.com) |
|  |  |

**SIGNATURE PAGE**

The signature below documents the review and approval of this protocol and provides the necessary assurances that this study will be conducted according to the protocol, including all statements regarding confidentiality, and according to national, regional, and local legal and regulatory requirements.

____________CHUNYAN LI___________________

Site Principal Investigator Name (Print)

____________________________________________

Signature

____________________________________________

Date

**List of Abbreviations**

| PrEP | Pre-exposure prophylaxis |
| --- | --- |
| MSM | Men who have sex with men |
| RCT | Randomized controlled trial |
| STI | Sexually transmitted infections |
|  |  |
|  |  |

**Protocol Summary**

| Protocol Title | The Development of a Mobile-based App to Increase Uptake of Pre-Exposure Prophylaxis (PrEP) by Men Who Have Sex with Men in China: Study Protocol |
| --- | --- |
| Study Design | The study consists of a formative research phase to develop a mobile phone-based intervention, the mini-app, to increase individual knowledge, understanding and initiation of pre-exposure prophylaxis (PrEP) among HIV-negative adult men who have sex with men (MSM) in Guangzhou, China, followed by a two-arm randomized control trial to test its efficacy. |
| Study Population | Individual who reported aged 18 and above, assigned male sex at birth, ever had anal sex with another man, HIV-negative, identifying as Chinese citizen, currently residing Guangzhou(only applicable in Aim 2), China, and able to and being willing to sign written informed consent and participate in the study as procedures required. |
| Number of subjects | Total sample size: N=110  **Aim 1** (in-depth interviews): n=40  **Aim 2** (two-arm RCT): n=70 (intervention arm n=40, control arm n=30) |
| Number of sites | 1 |
| Estimated Start of Enrollment | June 15, 2020 |
| Estimated Time to Completion | June 15, 2021 |
| Protocol Duration | **Aim 1**: 90 minutes  **Aim 2**: **Aim 2**: 16 weeks (8-week active intervention and after-intervention follow-up at the 12^th^ week. Participants in the intervention arm can have access to the intervention app up to 16 weeks.)  Participants may choose to participate in either Aim 1 or Aim 2. |
| Stratification | Participants in Aim 1 will be stratified into three groups:  (1) 5 current PrEP users  (2) 30 people who have never used PrEP  (3) 5 intermittent PrEP users (people who were on PrEP before but now off) |
| Regimen or Intervention | **Aim 1**: Formative research/in-depth interviews: no intervention  **Aim 2**: Two-arm RCT: 70 participants will be randomized at enrollment into the intervention arm (n=40) or the standard of care control arm (n=30). All 70 participants will pay an initial visit to the Guangzhou No.8 People’s Hospital to complete enrollment and a Web-based baseline assessment survey.   - **The standard of care control arm**: during the initial visit, participants will receive written HIV prevention materials including basic facts of PrEP, recommendations for HIV/STIs testing and referrals to local HIV/STIs testing sites and prevention services. - **The intervention arm**: In addition to the standard of care, participants in the intervention arm will have access to a mini-app-based PrEP intervention for up to 16 weeks. The mini-app has four main functions: (1) a mini-classroom function that contains a series of HIV-, sexual health-, and PrEP-related educational articles in Chinese; (2) an HIV/Syphilis home-based test kit ordering system that allows users to order a finger-prick HIV rapid test toolkit and shipped to home for free (one piece at a time); (3) an asynchronous message function that allows users to communicate with the study team; and (4) a user profile page where users can manage orders of HIV test kits. |

Table of Contents

[1.0 OBJECTIVE 9](#_Toc50059719)

[1.1 Hypothesis 9](#_Toc50059720)

[1.2 Primary Objective 9](#_Toc50059721)

[1.3 Secondary Objectives 9](#_Toc50059722)

[2.0 INTRODUCTION 9](#_Toc50059723)

[2.1 Background 9](#_Toc50059724)

[2.2 Rationale 10](#_Toc50059725)

[3.0 STUDY DESIGN 11](#_Toc50059726)

[4.0 SELECTION AND ENROLLMENT OF PARTICIPANTS 16](#_Toc50059727)

[4.1 Inclusion Criteria 16](#_Toc50059728)

[4.2 Exclusion Criteria 16](#_Toc50059729)

[4.3 Study Enrollment Procedures 17](#_Toc50059730)

[5.0 STUDY TREATMENT (OR INTERVENTION) 17](#_Toc50059731)

[6.0 DATA COLLECTION 19](#_Toc50059732)

[6.1 Data collection activities 19](#_Toc50059733)

[6.2 Data Management 20](#_Toc50059734)

[6.3 Quantitative Outcome Measures 20](#_Toc50059735)

[6.4 Incentives 20](#_Toc50059736)

[7.0 PARTICIPANT WITHDRAW AND REPORT 21](#_Toc50059737)

[8.0 STATISTICAL CONSIDERATIONS 22](#_Toc50059738)

[8.1 General Design 22](#_Toc50059739)

[8.2 Endpoints 22](#_Toc50059740)

[8.3 Randomization and Stratification 22](#_Toc50059741)

[8.4 Sample Size and Accrual 22](#_Toc50059742)

[8.5 Monitoring 22](#_Toc50059743)

[8.6 Analyses 23](#_Toc50059744)

[9.0 STUDY MANAGEMENT 23](#_Toc50059745)

[9.1 Institutional Review Board (IRB) Review and Informed Consent 23](#_Toc50059746)

[9.2 Criteria for Discontinuation 24](#_Toc50059747)

[9.3 Obligations of Investigators 25](#_Toc50059748)

[9.4 Participant Confidentiality 26](#_Toc50059749)

[9.5 Study Discontinuation 26](#_Toc50059750)

[10.0 BIOHAZARD CONTAINMENT 26](#_Toc50059751)

[References 27](#_Toc50059752)

# 1.0 OBJECTIVE

### 1.1 Hypothesis

We hypothesize that in our study sample, adult men who have sex with men (MSM) in the intervention arm are more likely to report successful PrEP initiation at the end of trial, as compared to the control arm.

### 1.2 Primary Objective

Our primary objectives include:

(1) Conduct formative research to understand key barriers and facilitators for PrEP uptake among Chinese MSM and test the usability of a mobile-based mini-app intervention.

(2) Conduct a two-arm pilot randomized control trial to test the efficacy of the mini-app in increasing PrEP uptake rates among Chinese adult MSM.

### 1.3 Secondary Objectives

Secondary objectives include: in our study sample,

(1) Test the efficacy of the mini-app in decreasing PrEP stigma and increasing PrEP use self-efficacy at the individual level.

(2) Identify the predictors associated with successful PrEP initiation during the study period.

(3) Investigate the association between depression at baseline and successful PrEP initiation during the study period.

(4) Assess PrEP use adherence among participants who successfully started PrEP during the study period.

# 2.0 INTRODUCTION

### 2.1 Background

The prevalence of HIV among Chinese men who have sex with men (MSM) has steadily increased for the past decade. According to the National HIV Sentinel Surveillance System, the prevalence of HIV in Chinese MSM rose from 1.5% in 2005 to 8.0% in 2015.^1^ Overall, MSM comprise over a quarter of new HIV diagnoses in China, this greater than three times the rate in other countries.^2^ These trends continue to rise as China works to implement feasible and sustainable HIV prevention measures.

Pre-exposure prophylaxis (PrEP) is a once daily oral fixed-dosed combination of two antiretroviral medications, tenofovir disoproxil fumarate (TDF) and emtricitabine (FTC) also referred to as TDF/FTC. Several clinical trials have demonstrated that PrEP is safe and significantly reduces the rate of HIV acquisition in MSM,^3^ heterosexual HIV sero-discordant couples,^4^ heterosexual individuals,^5^ and persons who inject drugs.^6^ Based on these studies, in 2014, the United States Centers of Disease Control and Prevention (CDC) issued recommendations for PrEP in MSM, high risk heterosexual men and women, and PWID. These recommendations were updated in 2017 with more trials further supporting PrEP use in these key populations.^7^ In 2014, the World Health Organization (WHO) recommended global access to PrEP for MSM which was then expanded in 2015 to include all populations at substantial risk of HIV infection.^8^

Despite the current HIV landscape of China and the proven safety and efficacy of PrEP, uptake remains limited with only an estimated 400-600 current PrEP users in the country.^9^ The delay in PrEP expansion in China is likely multifactorial, including limited awareness of PrEP, HIV and sexuality-related stigma and discrimination, concerns of PrEP effectiveness and side effects, and financial concerns to afford PrEP.^10–13^ Initial PrEP demonstration projects in China were unsuccessful because of poor community engagement and lack of community ownership.^11^ Bottom-up approaches such as challenge contests provide a tool for MSM to be more actively engaged in HIV service delivery.^14^ A recent multisite survey of urban Chinese MSM showed while nearly half of the respondents endorsed unprotected anal sex, less than a quarter had heard of PrEP.^15^ These studies indicate a need in using an MSM-friendly approach to promote PrEP uptake among this at-high-risk population and linking them to PrEP to help stem the ongoing epidemic.

Mobile health (mHealth) intervention tools have become increasingly popular in HIV- and PrEP-related research. Formative studies indicate that mHealth interventions are acceptable and effective in promoting HIV prevention practices in MSM and other young populations.^16,17^ Among the few mHealth-based PrEP intervention studies, text messaging has been effective in improving PrEP adherence in MSM via reducing missed doses^18^ and increasing drug concentrations in blood.^19^ More mHealth PrEP uptake intervention studies are in the pipeline however all are based in the US.^20–23^ mHealth shows promise as an intervention tool in places like China where HIV risk factors and infection remains highly stigmatized.^24^ HIV-related and sexual health interventions delivered through Internet-enabled platforms are feasible and acceptable in Chinese settings,^25^ including interventions through websites, text message and mobile apps which have shown effectiveness in reducing HIV-related risk behaviors, increasing linkage to care, and medication adherence.^26–28^

### 2.2 Rationale

While most of our previous knowledge about the acceptability of PrEP in China came from cross-sectional survey studies, little is known from a qualitative perspective about the key concerns and thoughts that Chinese MSM are having of PrEP as an HIV prevention strategy. Further, either globally or in China, limited data exists on the efficacy of app-based interventions aimed to increase PrEP uptake and adherence. Given the gap in knowledge, this proposed study aims to deeply understand the key barriers and facilitators to PrEP uptake among Chinese MSM and their unmet needs in HIV prevention via in-depth interviews, followed by a two-arm pilot randomized controlled trail to test the efficacy of a mini-app in increasing their engagement for PrEP education and initiation. A prototype of the mini-app has been developed from an MSM-friendly Doctor Finder hackathon contest (April 2019, Guangzhou) by a team of Chinese young MSM. Our study will provide preliminary data for a competitive R01 to fully develop and test this new evidence-based intervention to increase PrEP knowledge and actual uptake in China. If successful, this research will ultimately impact the PrEP/HIV prevention cascade in China by showing that an mHealth intervention can alleviate systemic and psychosocial barriers to HIV prevention services among young Chinese MSM. This research addresses a critical need as MSM bear a disproportionate burden of China’s HIV infections and remain underserved in the healthcare system.

# 3.0 STUDY DESIGN

This study is designed to collect formative data and preliminary efficacy test result of a mini-app that will help inform a future full-scale intervention study to increase PrEP uptake in China. It consists of a formative phase using in-depths interviews (Aim 1, see Figure 1) and an experimental phase using a two-arm randomized controlled trial (RCT) design (Aim 2, see Figure 2).

**Aim 1: Understand the key barriers and facilitators of using PrEP, and access the usability and acceptability of a mobile-based intervention mini-app for PrEP education and initiation among MSM in China via in-depth interviews with 40 HIV-negative young Chinese MSM (18 years old and above) in China.**

In Aim 1 we will assess unmet needs in HIV prevention, key barriers and facilitators of PrEP uptake among HIV-negative young MSM in China. We will conduct in-depth interviews with 40 HIV-negative MSM including 30 people who have never used PrEP, 5 people who used PrEP before but are now off treatment, and 5 people who are current PrEP users. All potential participants will be verbally screened for eligibility during their initial contact with the study team. During the interview, participants will be asked to test use a mini-app prototype for 5 minutes on their own smartphone, after a brief instruction on the purpose of the mini-app and how to use it. The mini-app is built in an existing popular social networking app (WeChat) that any user with a test link will be able to access the mini-app via WeChat without downloading additional installments. Then participants will be asked questions about their perceived feasibility and acceptability of the mini-app. A brief demographic survey will be administrated at the end of interview. The mini-app has four main functions: (1) a mini-classroom that contains a series of HIV-, sexual health-, and PrEP-related educational articles in Chinese; (2) an HIV/Syphilis duo self-test toolkit ordering system that allows users to order a finger-prick HIV rapid test toolkit and shipped to home for free (one kit per order); (3) an asynchronous message function that allows users to initiate a chat with a study staff; and (4) a user profile page where users can manage all HIV test orders.

Interviewees’ demographics and PrEP use history will be recorded in a spreadsheet in order to balance enrollment across all 3 groups (current PrEP users, people who never use PrEP, and intermittent PrEP users). We will use semi-structured interview guides with tailored questions for each group of the participants (see attached in-depth interview guide). In addition to discussing their knowledge, attitudes and willingness to use PrEP and PrEP use history, interviewees will be asked to describe their past pathways to HIV prevention services (e.g., HIV testing, counseling) including struggles and support resources they encountered. After test-using the mini-app, participants will be asked to comment on the design, contents and ease of use of the mini-app. Interviews will be conducted one-on-one in private spaces, last 60-90 minutes and will be audio recorded with participant's permission.

Interview: Feedback on the mini-app, e.g., using experience, designs and contents, relevance, and suggestions for scaling up PrEP in China.

Participant Complete a short app usability survey

Interview: Past experience and needs in HIV prevention and care, and perceptions of taking medicines to prevent HIV (such as PrEP)

Participant test use the mini-app

(5-10 mins)

Figure 1. Procedure of in-depth interview in Aim 1

**Aim 2: Evaluate the preliminary efficacy of the mini-app in changing PrEP perceptions and increasing PrEP uptake among young Chinese MSM (18 years old and above) in Guangzhou, China through a small pilot RCT (n=70), comparing the mini-app (n=40) to the standard of PrEP care (n=30).**

Before the start of Aim 2, we will refine the mini-app based on participants’ feedback in Aim 1. In Aim 2, we will conduct a pilot two-arm RCT to evaluate the feasibility and acceptability of the mini-app and examine its preliminary efficacy in changing PrEP-related perceptions and increasing actual PrEP initiation compared with a standard of care control group. All potential participants who show interest in participating the study need to complete a verbal eligibility screening during their initial contact with the study team. Those screened eligible and interested in participation will need to make an initial in-person or remote visit at the Guangzhou No.8 People’s Hospital, during which they complete a baseline assessment survey after proceeding the full informed consent process. Only participants who express interest in using the mini-app for HIV prevention, meet the eligibility criteria, provide informed consent, and complete a baseline assessment will be assigned a final study ID and be eligible for randomization.

Control: Standard of Care

In the standard of care control arm, participants will receive written HIV prevention materials (hard-copy at the clinic or electronic files) including basic facts of PrEP, recommendations for HIV/STIs testing and referrals to local HIV/STIs testing sites and prevention services.

Intervention: the mini-app

The study intervention tool, a mini-app built within a chat-app (WeChat, available on both Android and iOS systems), serves as the primary participant-facing component of the intervention. The mini-app does not require users to download or install anything as long as they have WeChat installed on their phone. Participants in the intervention arm will be able to access full function of the mini-app for 8 weeks. The mini-app has four main functions: (1) a mini-classroom that contains a series of HIV-, sexual health-, and PrEP-related educational articles in Chinese; (2) an HIV/syphilis home-based test kit ordering system that allows users to order a finger-prick HIV rapid test toolkit and ship to home for free (one toolkit per order allowed; participants can place more than one orders with no upper limit during the intervention period); (3) an asynchronous message function that allows users to communicate with the study team; and (4) a user profile page where users can manage orders of HIV/syphilis test kits. The mini-app is not able to collect or track any individual user information or user activity within the mini-app. It cannot initiate a chat with participants unless the participant requests a chat.

Home-based HIV/Syphilis test kit

The study will use a mail-based system to deliver free HIV/Syphilis test kits to participants upon request. We will use imported Abbott SD BIOLINE HIV/Syphilis Duo test kit that allows users to conduct finger-prick test at home. Participants will receive a plain box via standard mail that includes the test kit with instructions. Participants can also find the instruction of how to use the test kit in the mini-app. Participants do not need to send back any specimen or other material back to the study team, but they will be encouraged to report back test results to the study team via messaging through the mini-app. Testing experience and results will also be asked in the biweekly follow-up surveys.

PrEP initiation

Participants in both arms can choose to initiate PrEP at any time point since the initial visit until the end of 8^th^ week. Participants can contact the study team via phone call, text messages or via the chat function in the mini-app to communicate their interest in PrEP initiation. If baseline assessment or follow-up survey results do not show contradictions to PrEP initiation, interested participants will be referred to the Department of Infectious Diseases at Guangzhou No.8 People’s Hospital to consult a clinician regarding HIV risks and PrEP eligibility. As is standard of care for any patient starting PrEP, participants will take comprehensive physical examinations (including routine urine examinations, hepatic and renal function tests, blood glucose and lipids, and bone mineral density (BMD)). The study team will not have access to participants’ medical records (i.e. the physical examination results). Participants who are confirmed by the clinician HIV-negative, with no serious liver or kidney dysfunction and negative for HBs antigen, without serology indicating osteoporosis, without clinical signs or symptoms consistent with acute viral infection, will be prescribed either 8-week daily oral TDF/FTC or event-driven oral TDF/FTC based on participants’ preference. The cost of physical examinations in order to get PrEP prescription will be covered by the study team. If a participant is eligible for starting PrEP and have been prescribed PrEP by a PrEP provider, the participant will get a 50% reimbursement of the cost for their first two months of PrEP doses (30 pills/month, up to 60 pills in total) filled at the in-house pharmacy of the study hospital (the Guangzhou Eighth People’s Hospital).

Follow-up and Data collection

The active intervention period will last for 8 weeks, but participants in the intervention arm can have access to the mini-app throughout the whole study period or until the end of their first two-month PrEP use, which can be as long as 16 weeks. Baseline assessments will be conducted at the enrollment visit, with follow-up assessments conducted at 2, 4, 6, 8, 12 (post-intervention). Participants from both arms will need to complete a self-administrated Web-based survey at each assessment time point.

In addition to Web-based surveys, a subgroup of participants (n=20) from both the intervention arm and the control arm will receive up to two rounds of in-depth interviews, separately at the 4th and 8th week, to share their experience of using the app and changed perceptions and/or behaviors related to PrEP. Among the 20 participants, 15 will be recruited for interviews during the first and fourth weeks, who will receive two rounds of interviews. Another five participants will be recruited between the 4th and 8th week, who will be only interviewed once at the 8th week. Interview topics include participants’ experience of using the app and changed perceptions and/or behaviors related to PrEP and HIV prevention practices during the study period. Interviews will be conducted one-on-one in private spaces, last 60-90 minutes and will be audio recorded with participant's permission.

Referrals

In the case of an initial positive HIV test done through the study, participants who have initiated PrEP will be instructed to discontinue PrEP dosing. Participants testing positive will be referred to the Guangzhou No.8 People’s Hospital for confirmation test or other testing places if needed. The Guangzhou Center for Diseases Prevention and Control will be notified of confirmed positive results in accordance with China’s public health reporting laws, a procedure that will be explained to participants at consent. For positive syphilis testing results, participants will be referred to STI treatment at the Guangzhou No.8 People’s Hospital. The study team will follow-up with participants testing positive for HIV or STIs to encourage participants to seek appropriate care.

Eligibility screening over phone/online survey

Participant Recruitment

PrEP prescription (8-week doses)

No.8: Physical examinations

Participants interested in PrEP initiation

During the first 8-week period

8-week

intervention

4^th^ week in-depth interview

(10 from the app arm +

5 from the control arm)

8^th^ week survey

6^th^ week survey

4^th^ week survey

2^nd^ week survey

Standard of care arm (n=20)

Mini-app arm (n=40)

Initial visit at Guangzhou No.8 People’s Hospital

(Full consent procedure & **baseline assessment**)

8^th^ week in-depth interview

(same 15 participants from 4^th^ week + another 5 new participants)

After

intervention

12^th^ week survey

Figure 2. Flowchart of Aim 2 study design

# 4.0 SELECTION AND ENROLLMENT OF PARTICIPANTS

### 4.1 Inclusion Criteria

**Aim 1** (in-depth interviews): 40 HIV-negative men who have sex with men (MSM): must self-report: being assigned male sex at birth, HIV-negative, age 18 and above, ever having had anal sex with another man, currently reside in Guangzhou, China, identify as a Chinese citizen, able to being willing to sign written informed consent and participate in the study as procedures required.

**Aim 2** (randomized controlled trial, RCT): 70 HIV-negative MSM: same characteristics as in Aim 1. AND, they must report:

- Having a smartphone with WeChat installed.
- Being assigned male sex at birth, HIV-negative, age 18 and above, ever having had anal sex with another man, currently reside in Guangzhou, China, identifying as a Chinese citizen, able to sign written informed consent and participate in the study as procedures require. AND
- Not currently using any medicine as PrEP
- At least one criterion associated with high risk of HIV infection in the past 6 months prior to enrollment as follows:
- Unprotected (condomless) anal intercourse with male partner(s)
- More than two male partners (regardless of condom use and HIV serostatus)
- Reported STI, such as syphilis, HSV-2, gonorrhea, chlamydia, chancroid, or lymphogranuloma venereum.
- Reported use of post-exposure prophylaxis (PEP)
- Have a sexual partner living with HIV

### 4.2 Exclusion Criteria

**Aim 1**: Individuals who report any following status will be excluded from participating Aim 1:

- HIV-positive
- Mental health issues which may compromise participant adherence or safety, including memory loss, cognitive impairment, intellectual disability, or communication disorders.

**Aim 2**: Individuals who report any following status will be excluded from participating Aim 2

- HIV-positive
- Currently taking oral PrEP based on self-report
- Symptoms of acute HIV infection within the prior 30 days
- Contradictions to taking oral PrEP
- Personal diagnosis or family history of hemophilia Chronic Hepatitis B (self-report)
- Participating in another research study related to HIV and antiretroviral therapy or other intervention study
- Having serious chronic disease, including metabolic diseases (such as diabetes), neurological, and psychiatric disorders
- Mental health issues which may compromise participant adherence or safety, including memory loss, cognitive impairment, intellectual disability, or communication disorders.

### 4.3 Study Enrollment Procedures

Participant recruitment will be targeted to venues (health clinics, gay bar) and mediums (e.g. Internet, social media) that we have identified from past formative work to be frequented by MSM in Guangzhou, and specifically MSM who are seeking HIV testing or post-exposure prophylaxis.

**Aim 1**: Potential participants will be verbally screened for eligibility during their initial contact with the study team, before proceeding to the full informed consent and enrollment in in-depth interviews. All enrolled participants will be given a unique ID number for identification purposes.

**Aim 2**: Potential participants will be verbally screened for eligibility during their initial contact with the study team, before proceeding to the full informed consent and enrollment in the RCT. All enrolled participants will be given a unique ID number for identification purposes.

We will conduct a simple randomization that randomly assign the 70 participants to either the mini-app arm (n=40) or the control arm (n=30). Based on the timing of enrolling the study, each participant will be assigned a two-digit study ID (e.g., the first enrolled participant will be ID01; the second will be ID02; the last one enrolled will be ID70). Before enrollment, the study team will use the Microsoft Excel program to randomly assign the 70 IDs into group A (the intervention arm) and group B (the control arm). Once enrolled, a participant will be automatically assigned to either the intervention arm or the control arm based on his ID. If a participant quitted the study because of non-PrEP-related reasons (e.g., seroconversion, moving to other cities) after enrollment, we will recruit a new participant for replacement.

# 5.0 STUDY TREATMENT (OR INTERVENTION)

**Aim 1**: No study treatment or intervention.

**Aim 2**: Participants will receive two types of study treatment:

(1) Standard of Care

During their initial visit at Guangzhou No.8 People’s Hospital (in-person or via videoconferencing systems), after enrollment, participants from both arms will receive written HIV prevention materials (hard-copy at the study clinic or electronic files) including basic facts of PrEP, recommendations for HIV/STIs testing and referrals to local HIV/STIs testing sites and prevention services.

(2) The Mini-app Intervention

In addition to the standard of care, participants who are assigned to the intervention arm will receive a mini-app-based PrEP intervention for up to 16 weeks. The intervention tool is a mini-app that is built in a popular Chinese social media mobile platform, WeChat, to increase the engagement for PrEP education and PrEP initiation among Chinese MSM.

Right after enrollment, participants randomized to the intervention arm will be guided by study staff through the mini-app access process and given a tour to highlight features. A prototype of the mini-app has been developed from an MSM-friendly doctor finder hackathon contest (April 2019, Guangzhou) by a team of Chinese young MSM. The mini-app will be refined in terms of prioritized topics, graphical designs and tones of language based on results from Aim 1 before starting trial in Aim 2. The overall goal of the mini-app is for PrEP education. It has four main functions: (1) a mini-classroom that contains a series of HIV-, sexual health-, and PrEP-related educational articles in Chinese; (2) an HIV/syphilis self-test toolkit ordering system that allows users to order a finger-prick HIV rapid test toolkit and shipped to home for free (one piece at a time); (3) an asynchronous message function that allows users to chat with a trained PrEP counselor; and (4) a user profile page where users can manage all HIV test orders. The mini-app wireframe is presented in Figure 3.


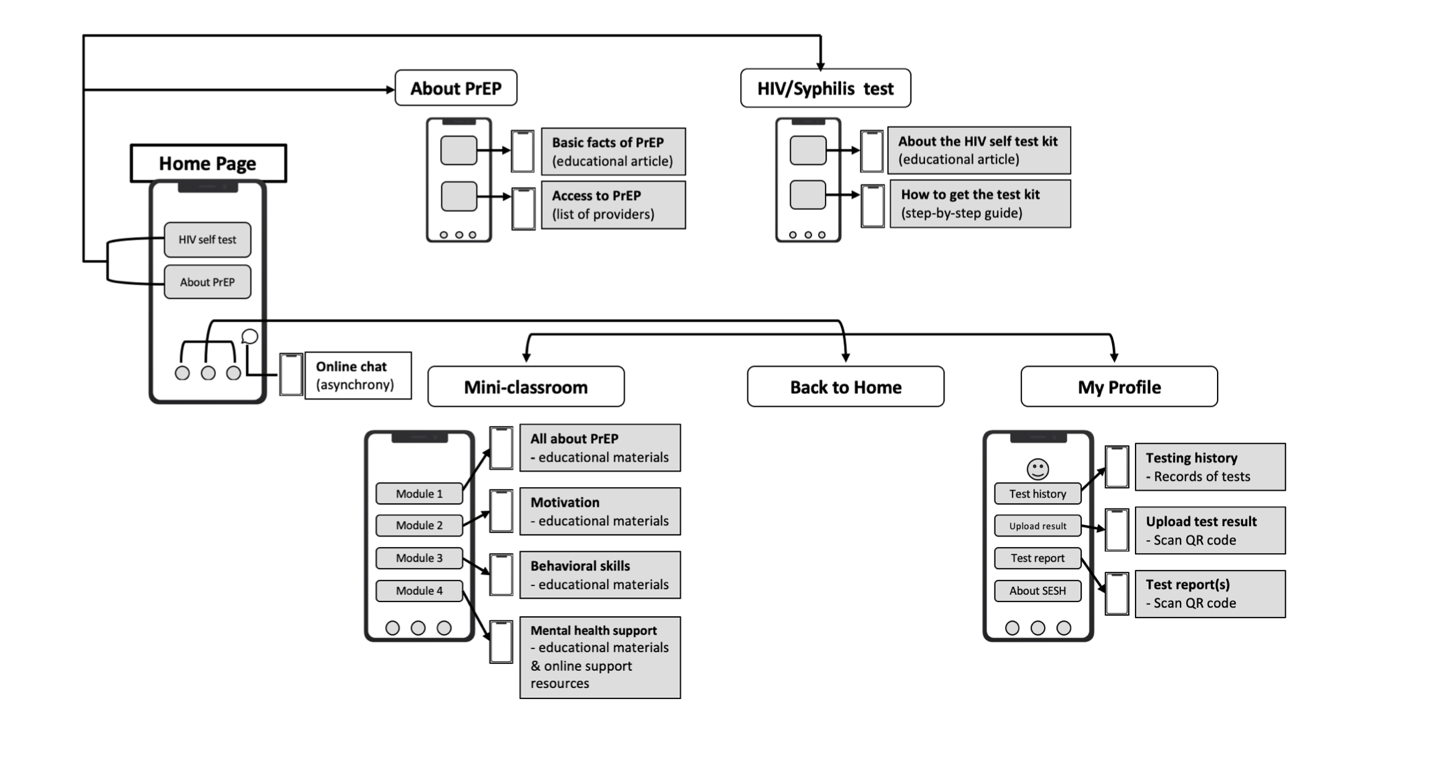


Figure 3. Wireframe of the mini-app PrEP intervention (Aim 2)

Participants in the intervention arm will have access to the mini-app for up to 16 weeks since enrollment. Usage of the mini-app will at participants’ own discretion or preference. A one-sentence weekly reminders that encourage participants to use the mini-app will be sent out through text messages. The mini-app will not be able to track any individual user information or user activity within the mini-app. Self-reported app usage will be assessed in bi-weekly follow-up surveys and in-depth interviews. At 8^th^ week, participants in the intervention arm will be allowed to continue using the mini-app, but will not no longer receive reminder messages.

# 6.0 DATA COLLECTION

### 6.1 Data collection activities

Data collection activities will be described by study aims.

**Aim 1**: Qualitative interviews

One-on-one in-depth interviews will be conducted in multiple settings in Guangzhou, China including an HIV testing clinic, and private locations of the participants' choosing (e.g. private room in a tea shop, private office space). Each interview will last 60-90 minutes. Interviews will be audio taped, transcribed verbatim in Chinese, summarized in English for analysis. During the interview participants will also be required to complete a 5-point Likert usability scale and a brief demographic survey.

Due to the social distancing requirement during the COVID-19 pandemic as well as to reduce health risks for both participants and research staff, in-depth interviews may be conducted via videoconferencing system (e.g., Zoom, WeChat video/audio call) depending on the participant’s preference.

**Aim 2**: Two-arm RCT

Data collection in Aim 2 include Web-based baseline and follow-up assessment surveys, and qualitative interviews.

Web-based surveys

Baseline assessment will be conducted at the initial enrollment visit, with follow-up assessments conducted at 2, 4, 6, 8, and 12 weeks. All Web-based survey will be self-administrated and collected through a Chinese professional secure electronic survey platform (www.wjx.cn). Survey links will be sent to participants via text message that participants can choose to finish the survey on their phone or computer.

Qualitative interview

Similar to in-depth interviews in Aim 1, in-depth interviews in Aim 2 will be conducted in multiple settings in Guangzhou, China including an HIV testing clinic, and private locations of the participants' choosing (e.g. private room in a tea shop, private office space).

Due to the social distancing requirement during the COVID-19 pandemic as well as to reduce health risks for both participants and research staff, in-depth interviews and enrollment visit may be conducted via videoconferencing system (e.g., Zoom, WeChat video/audio call) depending on the participant’s preference.

### 6.2 Data Management

Only authorized users (PIs, faculty advisor), based on correct login will be able to access and open the study surveys and in-depth interview recordings and transcripts. In-depth interviews will be audio taped, transcribed verbatim (in Chinese), summarized in English and organized and managed using Dedoose cloud-based qualitative data analysis software ([www.dedoose.com](http://www.dedoose.com)). Web-based survey will be collected through a Chinese professional secure electronic survey platform Wenjuanxing ([www.wjx.cn](http://www.wjx.cn)). Survey data will be downloaded from Wenjuanxing and will be stored on password protected encrypted study computers along with other electronic study files. All study files will have a back-up copy stored on UNC secure server space that only study personnel will have access to. All information will be kept confidential, with responses labeled by an identification number available only to investigators in locked or password secured files.

### 6.3 Quantitative Outcome Measures

Quantitative outcome measures will be assessed in Aim 2 through baseline and follow-up assessments.

**Primary Outcome Measures**: The primary outcome is PrEP initiation, measured by the number of participants who successfully initiated PrEP during the 8-week intervention period.

**Secondary Outcome Measures**: Secondary outcomes include self-report PrEP use self-efficacy, PrEP stigma, PrEP adherence (among those who are on PrEP), sexual risk behaviors, sexually transmitted infection incidence, general stress and HIV-related stress.

### 6.4 Incentives

**Aim 1**: Remuneration will be provided at the end of each completed interview (total of three possible interviews) in the form of a 75-CNY (~ 10 USD) gift card or equivalent. The participant may opt to stop the interview at any time, however remuneration will only be provided for completed interviews.

**Aim 2**: Participants in Aim 2 will receive up to 340 CNY in total, including a 50-CNY (~ 7 USD) gift card for the in-person initial visit or baseline assessment at Guangzhou No.8 People’s Hospital, and another 20-CNY (~3 USD) gift card for completing each Web-based long follow-up survey at weeks 4, 8, and 12, and 5-CNY (~1 USD) gift card for complete each Web-based short survey at weeks 2 and 6. Participants who are selected for in-depth interviews will receive 75-CNY (~10 USD) gift card for completing each interview (two interviews in total). Remuneration will be provided at the end of each completed session. The participant may opt to stop the interviews/baseline assessment/follow-up surveys at any time, however remuneration will only be provided for completed sessions. Participants who participate in all the required research activities will receive a bonus incentive of 50 CNY at the end of the study.

# 7.0 PARTICIPANT WITHDRAW AND REPORT

Participants may voluntarily withdraw from the study for any reason at any time. No further data collection will occur from the date the decision is made to permanently discontinue the participant from the study. The study investigators also may withdraw participants from the study in order to protect participant or staff safety. Study staff will record the reasons for all withdrawals.

If a participant fails or is unable to comply with study procedures they will be withdrawn from the study. Participants will be withdrawn if they:

- are noticeably intoxicated or on illegal drugs.
- threaten a study staff member.
- are mentally handicapped and unable to give full informed consent.
- are incarcerated during course of study.
- become HIV-positive during course of study.
- reporting severe medication side effects after initiating PrEP and are advised to quit the study by a HIV specialist.

Any enrolled participant who report any of the above-mentioned events will be immediately stop participation in the study, and study staff will complete a Study Stop form for participants who are discontinued. This form will include the date, last research activity completed, and reason for study discontinuation. These will be reported to the UNC IRB and the Guangzhou No.8 People’s Hospital IRB at the end of study.

Reporting is required of all unanticipated problems to the UNC IRB, including those which may occur after the participant has completed or has withdrawn from the study, including after study closure.

Unanticipated problems as defined by the UNC IRB:

Unanticipated problems involving risks to participants or others” or “Unanticipated Problem” refers to any incident, experience, or outcome that:

- is unexpected (in terms of nature, severity, or frequency) given (a) the research procedures that are described in the protocol-related documents, such as the IRB-approved research protocol and informed consent document; and (b) the characteristics of the participant population being studied;
- is related or possibly related to a participant’s participation in the research; and
- suggests that the research places participants or others at a greater risk of harm (including physical, psychological, economic, or social harm) related to the research than was previously known or recognized.

Unanticipated Problems that are serious adverse events should be reported to the UNC IRB ang Guangzhou No.8 People’s Hospital IRB within one (1) week of the investigator becoming aware of the event. Any other Unanticipated Problem should be reported to the UNC IRB and Guangzhou No.8 People’s Hospital IRB within two (2) weeks of the investigator becoming aware of the problem. If the Unanticipated Problem Report cannot be completed in its entirety within the required time period, a preliminary report should be submitted. The Unanticipated Problem Report should be amended once the event is resolved and/or more information becomes available.

# 8.0 STATISTICAL CONSIDERATIONS

This section is only applicable to Aim 2.

### 8.1 General Design

Aim 2 include a two-arm unblinded RCT, testing the efficacy of a mini-app-based PrEP intervention in promoting PrEP uptake among Chinese MSM compared to standard of care control.

### 8.2 Endpoints

The endpoint for primary outcome is successful initiation of PrEP.

### 8.3 Randomization and Stratification

Participants will be simply randomized at initial enrollment visit into two arms (intervention=40, control=30). No stratification will be conducted.

### 8.4 Sample Size and Accrual

This is a pilot study to test the preliminary efficacy of a WeChat-based mini-app in promoting PrEP uptake rates in an 8-week intervention. Inclusion of a control arm will allow estimation of an effective size to sufficiently power a future full-scale efficacy trial. The full-size intervention study will be designed as a two-arm RCT, following participants up to 12 months and use multilevel modeling methods to understand the association between intervention exposure, changed PrEP perceptions, PrEP initiation and long-term adherence.

### 8.5 Monitoring

Data will be collected and stored by anonymous identification numbers by study investigators. Data will not be shared with third parties. Data will be discarded 2 years following study completion.

Plans for monitoring participant safety and study progress

The PIs will receive regularly bi-weekly updates from study personnel. In addition, study procedures are established that if at any time during the study a participant divulges that he is at risk for harm, including but not limited to reporting severe medication side effects, being abused or experiencing violence, if harm is suspected or likely, or if the participant states he is suicidal/homicidal, the PIs will be notified immediately and measures will be taken to ensure the participant's safety. Reporting will be done as appropriate to the situation and the legal statutes, and referrals will be provided for appropriate support, counseling, or treatment resources.

The full study team will hold bi-weekly conference calls to report progress on enrollment, data collection and data analysis. Any challenges that arise will be discussed during these calls and plans made to address.

This study is expected to pose minimal risk. Nevertheless, any unanticipated problems will be recorded in a master log by the PIs and reported during bi-weekly updates (or immediately as described above). The master log will be reviewed during bi-weekly full study team conference calls. Participants may voluntarily withdraw from the study for any reason at any time. Detailed description of participant withdraw and report can be found in section 7.0 in this protocol.

### 8.6 Analyses

Descriptive statistical analysis will be first conducted to report demographic characteristics of participants, PrEP uptake rates, PrEP-related perceptions, PrEP adherence, HIV/STI testing behaviors, prevalence of STIs, mini-app use behaviors at different time points throughout the study period. Statistical tests including Chi-Square tests and t-tests will be conducted to compare the preliminary intervention effects (i.e. the efficacy of mini-app in improving PrEP-related perceptions, increasing PrEP uptake rates and HIV/STI testing behaviors) between the intervention group and control group at each time point. Multivariate logistic regression models will be applied to estimate the association between intervention arm and the primary study outcome, PrEP initiation during the 8-week intervention period. If prognostic factors associated with the primary outcome remain insufficiently balanced through simple randomization, we may adjust for these factors in our models. Potential confounding factors that may be included are perceived HIV infection risk, PrEP self-efficacy and perceived PrEP stigma.

# 9.0 STUDY MANAGEMENT

### 9.1 Institutional Review Board (IRB) Review and Informed Consent

This protocol and the informed consent documents and any subsequent modifications (amendments) will be reviewed and approved by the UNC IRB and Guangzhou No.8 People’s Hospital IRB that are responsible for oversight of the study.

It is expected that the IRB will have the proper representation and function in accordance with federally mandated regulations. The IRB should approve the consent form(s) and protocol.

In obtaining and documenting informed consent, the investigator should comply with the applicable regulatory requirement(s), and should adhere to Good Clinical Practice (GCP) and to ethical principles that have their origin in the Declaration of Helsinki.

Before recruitment and enrollment onto this study, the participant will be given a full explanation of the study and will be given the opportunity to review the consent form(s). Each consent form must include all the relevant elements currently required by the FDA Regulations and local or state regulations, which include elements such as the purpose of the study, the procedures to be followed, and the risks and benefits of participation. Once this essential information has been provided to the participant and the investigator is assured that the participant understands the implications of participating in the study, the participant will be asked to give consent to participate in the study by signing an IRB‑approved consent form.

Prior to a participant’s participation in the trial, the written informed consent form(s) should be signed and personally dated by the participant or the participant’s legally authorized representative (legal guardian or person with power of attorney for participants who cannot consent for themselves), and by the person who conducted the informed consent discussion.

A copy of the consent form will be given to the participant or legal guardian, and this fact will be documented in the participant’s record.

### 9.2 Criteria for Discontinuation

###### 9.2.3 Premature Study Discontinuation

- Request by the participant to withdraw
- Request of the primary care provider if s/he thinks the study is no longer in the best interest of the participant
- Participant judged by the investigator to be at significant risk of failing to comply with the provisions of the protocol as to cause harm to self or seriously interfere with the validity of the study results
- At the discretion of the ACTU, IRB, FDA, Office for Human Research Protections (OHRP), NIAID, investigator, or pharmaceutical supporters

###### 9.2.3 Deviations from the Protocol

The investigator may implement a deviation from, or a change of, the protocol to eliminate an immediate hazard(s) to study participants without prior IRB/IEC approval/favorable opinion. As soon as possible, the implemented deviation or change, the reasons for it, and, if appropriate, the proposed protocol amendment(s) should be submitted to:

a) IRB for review and approval

b) to the sponsor for agreement and, if required

c) to the regulatory authority(ies)

###### 9.2.4 Required Documentation

Before the study can be initiated at any site, the following documentation must be provided to the ACTU/CID/IGHID Office at the University of North Carolina.

- A copy of the official IRB approval letter for the protocol and informed consent(s)
- IRB membership list
- CVs for the principal investigator and any associate investigators who will be involved in the study
- A copy of the IRB‑approved consent form(s)

###### 9.2.5 Adherence to the Protocol

Except for an emergency situation in which proper care for the protection, safety, and well-being of the study participant requires alternative treatment, the study shall be conducted exactly as described in the approved protocol. Any deviation from the protocol must have prior approval by the Principal Investigator and must be recorded and explained.

###### 9.2.6 Amendments to the Protocol

Should amendments to the protocol be required, the amendments will be originated and documented by the Principal Investigator at UNC. The written amendment will be sent to investigators and must be submitted to the IRB at the investigator's site for approval. It should also be noted that when an amendment to the protocol substantially alters the study design or the potential risk to the participant, a revised consent form might be required.

###### 9.2.7 Record Retention

Study documentation includes all Case Report Forms, data correction forms or queries, source documents, Sponsor-Investigator correspondence, monitoring logs/letters, and regulatory documents (e.g., protocol and amendments, IRB correspondence and approval, signed participant consent forms).

Source documents include all recordings of observations or notations of clinical activities and all reports and records necessary for the evaluation and reconstruction of the clinical research study.

Government agency regulations and directives require that all study documentation pertaining to the conduct of a clinical trial must be retained by the study investigator. In the case of a study with a drug seeking regulatory approval and marketing, these documents shall be retained for at least two years after the last approval of marketing application in an International Conference on Harmonization (ICH) region. In all other cases, study documents should be kept on file until three years after the completion and final study report of this investigational study.

### 9.3 Obligations of Investigators

The Principal Investigator is responsible for the conduct of the clinical trial at the site in accordance with Title 21 of the Code of Federal Regulations and/or the Declaration of Helsinki. The Principal Investigator is responsible for personally overseeing the treatment of all study participants. The Principal Investigator must assure that all study site personnel, including co-investigators and other study staff members, adhere to the study protocol and all FDA/GCP regulations and guidelines regarding clinical trials both during and after study completion.

The Principal Investigator at each institution or site will be responsible for assuring that all the required data will be collected and entered onto the Case Report Forms (CRFs). Periodically, monitoring visits will be conducted and the Principal Investigator will provide access to his/her original records to permit verification of proper entry of data. Site monitors will visit participating sites to review the individual participant records, including consent forms, CRFs, supporting data, laboratory specimen records, and medical records (physicians’ progress notes, nurses’ notes, individuals’ hospital charts), to ensure protection of study participants, compliance with the protocol, and accuracy and completeness of records. The monitors also will inspect sites’ regulatory files to ensure that regulatory requirements are being followed and sites’ pharmacies to review product storage and management. At the completion of the study, all case report forms will be reviewed by the Principal Investigator and will require his/her final signature to verify the accuracy of the data.

The site investigator will make study documents (e.g., consent forms, drug distribution forms, CRFs) and pertinent hospital or clinic records readily available for inspection by the local IRB, the site monitors, the FDA, the NIAID, the OHRP, and the pharmaceutical supporter or designee for confirmation of the study data.

### 9.4 Participant Confidentiality

All evaluation forms, reports, and other records that leave the site will be identified by coded number only to maintain participant confidentiality. All records will be kept locked. All computer entry and networking programs will be done with coded numbers only. Participant information will not be released without written permission of the participant, except as necessary for monitoring by the ACTU, IRB, the FDA, the NIAID, the OHRP, or the pharmaceutical supporters or designee.

### 9.5 Study Discontinuation

The study may be discontinued at any time by the ACTU, IRB, the NIAID, the pharmaceutical supporters, the FDA, the OHRP, or other government agencies as part of their duties to ensure that research participants are protected.

# 10.0 BIOHAZARD CONTAINMENT

No specimens will be collected or handled in this study.

# References

1. Tang S, Tang W, Meyers K, Chan P, Chen Z, Tucker JD. HIV epidemiology and responses among men who have sex with men and transgender individuals in China: A scoping review. *BMC Infect Dis*. 2016;16(1):1-9. doi:10.1186/s12879-016-1904-5

2. Peng P, Su S, Fairley CK, et al. A Global Estimate of the Acceptability of Pre-exposure Prophylaxis for HIV Among Men Who have Sex with Men: A Systematic Review and Meta-analysis. *AIDS Behav*. 2018;22(4):1063-1074. doi:10.1007/s10461-017-1675-z

3. Grant RM, Lama JR, Anderson PL, et al. Preexposure Chemoprophylaxis for HIV Prevention in Men Who Have Sex with Men. *N Engl J Med*. 2010;363(27):2587-2599. doi:10.1056/NEJMoa1011205

4. Baeten JM, Donnell D, Ndase P, et al. Antiretroviral prophylaxis for HIV prevention in heterosexual men and women. *N Engl J Med*. 2012;367(5):399-410. doi:10.1056/NEJMoa1108524

5. Thigpen MC, Kebaabetswe PM, Paxton LA, et al. Antiretroviral preexposure prophylaxis for heterosexual HIV transmission in Botswana. *N Engl J Med*. 2012;367(5):423-434. doi:10.1056/NEJMoa1110711

6. Choopanya K, Martin M, Suntharasamai P, et al. Antiretroviral prophylaxis for HIV infection in injecting drug users in Bangkok, Thailand (the Bangkok Tenofovir Study): A randomised, double-blind, placebo-controlled phase 3 trial. *Lancet*. 2013;381(9883):2083-2090. doi:10.1016/S0140-6736(13)61127-7

7. Centers for Disease Control and Prevention (CDC). *US Public Health Service: Preexposure Prophylaxis for the Prevention of HIV Infection in the United States - 2017 Update: A Clinical Practice Guideline*.; 2017. doi:10.1016/S0040-4039(01)91800-3

8. World Health Organization. *Guideline on When To Start Antiretroviral Therapy and on Pre-Exposure Prophylaxis for Hiv*.; 2015. doi:978 92 4 150956 5

9. China – PrEPWatch. https://www.prepwatch.org/country/china/. Published 2019. Accessed December 17, 2019.

10. Wang Z, Lau JTF, Yang X, et al. Acceptability of Daily Use of Free Oral Pre-exposure Prophylaxis (PrEP) Among Transgender Women Sex Workers in Shenyang, China. *AIDS Behav*. 2017;21(12):3287-3298. doi:10.1007/s10461-017-1869-4

11. Ding Y, Yan H, Ning Z, et al. Low willingness and actual uptake of pre-exposure prophylaxis for HIV-1 prevention among men who have sex with men in Shanghai, China. *Biosci Trends*. 2016;10(2):113-119. doi:10.5582/bst.2016.01035

12. Wang Z, Lau JTF, Fang Y, Ip M, Gross DL. Prevalence of actual uptake and willingness to use pre-exposure prophylaxis to prevent HIV acquisition among men who have sex with men in Hong Kong, China. *PLoS One*. 2018;13(2):1-18. doi:10.1371/journal.pone.0191671

13. Zheng ZW, Qiu JL, Gu J, Xu HF, Cheng W Bin, Hao C. Preexposure prophylaxis comprehension and the certainty of willingness to use preexposure prophylaxis among men who have sex with men in China. *Int J STD AIDS*. 2019;30(1):4-11. doi:10.1177/0956462418781142

14. Tucker JD, Fenton KA. Innovation challenge contests to enhance HIV responses. *Lancet HIV*. 2018;5(3):e113-e115. doi:10.1016/S2352-3018(18)30027-4

15. Han J, Bouey JZH, Wang L, et al. PrEP uptake preferences among men who have sex with men in China: results from a National Internet Survey. *J Int AIDS Soc*. 2019;22(2):1-9. doi:10.1002/jia2.25242

16. Cohen MS, Smith MK, Muessig KE, Hallett TB, Powers KA, Kashuba AD. Antiretroviral treatment of HIV-1 prevents transmission of HIV-1: Where do we go from here? *Lancet*. 2013;382(9903):1515-1524. doi:10.1016/S0140-6736(13)61998-4

17. Ko NY, Hsieh CH, Wang MC, et al. Effects of internet popular opinion leaders (ipol) among internet-using men who have sex with men. *J Med Internet Res*. 2013;15(2):1-10. doi:10.2196/jmir.2264

18. Fuchs JD, Stojanovski K, Vittinghoff E, et al. A Mobile Health Strategy to Support Adherence to Antiretroviral Preexposure Prophylaxis. *AIDS Patient Care STDS*. 2018;32(3):104-111. doi:10.1089/apc.2017.0255

19. Moore DJ, Jain S, Dubé MP, et al. Randomized Controlled Trial of Daily Text Messages to Support Adherence to Preexposure Prophylaxis in Individuals at Risk for Human Immunodeficiency Virus: The TAPIR Study. *Clin Infect Dis*. 2018;66(10):1566-1572. doi:10.1093/cid/cix1055

20. Bauermeister JA, Golinkoff JM, Horvath KJ, Hightow-Weidman LB, Sullivan PS, Stephenson R. A multilevel tailored web app-based intervention for linking young men who have sex with men to quality care (get connected): Protocol for a randomized controlled trial. *J Med Internet Res*. 2018;20(8). doi:10.2196/10444

21. Biello KB, Marrow E, Mimiaga MJ, Sullivan P, Hightow-Weidman L, Mayer KH. A mobile-based app (Mychoices) to increase uptake of HIV testing and pre-exposure prophylaxis by young men who have sex with men: Protocol for a pilot randomized controlled trial. *J Med Internet Res*. 2019;21(1):1-11. doi:10.2196/10694

22. Gamarel KE, Darbes LA, Hightow-Weidman L, Sullivan P, Stephenson R. The development and testing of a relationship skills intervention to improve HIV prevention uptake among young gay, bisexual, and other men who have sex with men and their primary partners (we prevent): Protocol for a randomized controlled trial. *J Med Internet Res*. 2019;21(1). doi:10.2196/10370

23. LeGrand S, Knudtson K, Benkeser D, et al. Testing the Efficacy of a Social Networking Gamification App to Improve Pre-Exposure Prophylaxis Adherence (P3: Prepared, Protected, emPowered): Protocol for a Randomized Controlled Trial. *JMIR Res Protoc*. 2018;7(12):e10448. doi:10.2196/10448

24. Liu J, Ma H, He Y-L, et al. Mental health system in China: history, recent service reform and future challenges. *World Psychiatry*. 2011;10(3):210-216. doi:10.1002/j.2051-5545.2011.tb00059.x

25. Muessig KE, Bien CH, Wei C, et al. A mixed-methods study on the acceptability of using eHealth for HIV prevention and sexual health care among men who have sex with men in China. *J Med Internet Res*. 2015;17(4):e100. doi:10.2196/jmir.3370

26. Mi G, Wu Z, Wang X, et al. Effects of a Quasi-Randomized Web-Based Intervention on Risk Behaviors and Treatment Seeking Among HIV-Positive Men Who Have Sex With Men in Chengdu, China. *Curr HIV Res*. 2015;13(6):490-496. http://www.ncbi.nlm.nih.gov/pubmed/26105555. Accessed September 14, 2017.

27. Ruan Y, Xiao X, Chen J, Li X, Williams AB, Wang hong. Acceptability and efficacy of interactive short message service intervention in improving HIV medication adherence in Chinese antiretroviral treatment-naïve individuals. *Patient Prefer Adherence*. 2017;Volume 11:221-228. doi:10.2147/PPA.S120003

28. Cheng W, Cai Y, Tang W, et al. *Providing HIV-Related Services in China for Men Who Have Sex with Men*. Vol 94.; 2016. doi:10.2471/BLT.15.156406
